# Pose2Sim: An End-to-End Workflow for 3D Markerless Sports Kinematics—Part 1: Robustness

**DOI:** 10.3390/s21196530

**Published:** 2021-09-30

**Authors:** David Pagnon, Mathieu Domalain, Lionel Reveret

**Affiliations:** 1 Laboratoire Jean Kuntzmann, Université Grenoble Alpes, UMR CNRS 5224, 38330 Montbonnot-Saint-Martin, France; lionel.reveret@inria.fr; 2 Institut Pprime, Université de Poitiers, CNRS UPR 3346, 86360 Chasseneuil-du-Poitou, France; mathieu.domalain@univ-poitiers.fr; 3 INRIA Grenoble Rhône-Alpes, 38330 Montbonnot-Saint-Martin, France

**Keywords:** markerless motion capture, sports performance analysis, kinematics, computer vision, openpose, opensim, deep learning, robustness

## Abstract

Being able to capture relevant information about elite athletes’ movement “in the wild” is challenging, especially because reference marker-based approaches hinder natural movement and are highly sensitive to environmental conditions. We propose Pose2Sim, a markerless kinematics workflow that uses OpenPose 2D pose detections from multiple views as inputs, identifies the person of interest, robustly triangulates joint coordinates from calibrated cameras, and feeds those to a 3D inverse kinematic full-body OpenSim model in order to compute biomechanically congruent joint angles. We assessed the robustness of this workflow when facing simulated challenging conditions: (Im) degrades image quality (11-pixel Gaussian blur and 0.5 gamma compression); (4c) uses few cameras (4 vs. 8); and (Cal) introduces calibration errors (1 cm vs. perfect calibration). Three physical activities were investigated: walking, running, and cycling. When averaged over all joint angles, stride-to-stride standard deviations lay between 1.7° and 3.2° for all conditions and tasks, and mean absolute errors (compared to the reference condition—Ref) ranged between 0.35° and 1.6°. For walking, errors in the sagittal plane were: 1.5°, 0.90°, 0.19° for (Im), (4c), and (Cal), respectively. In conclusion, Pose2Sim provides a simple and robust markerless kinematics analysis from a network of calibrated cameras.

## 1. Introduction

### 1.1. Overall Context

Kinematic analysis in sports is a difficult challenge that highlights the limits of classic marker-based methods [1]. Reflective marker-based camera systems are complex to set up, require specific lightning conditions, and are overall challenging to use outside of a lab. Markers may fall off the body due to sharp accelerations or sweat, as well as hinder the natural movement of athletes, which is likely to affect their warm-up and focus. While the accuracy of landmark location is claimed to be sub-millimetric in marker-based methods [2], marker placement is tedious, intrusive, prone to positioning variability from the operator [3], and subject to skin movement artifacts, especially on soft tissues. Della Croce et al. found out that inter-operator variations in marker placements range from 13 to 25 mm, which can propagate up to 10° in joint angle prediction [4,5]. Tissue artifacts account for up to a 2.5 cm marker displacement at the thigh, for example, which can cause as much as a 3° error in knee joint angles tissues [6,7]. Joint positions must be calculated explicitly in marker-based methods, which introduces more variability: these errors range up to 5 cm, which can contribute up to 3° of error in lower limb joint kinematics [8]. Nevertheless, since marker-based methods benefit from decades of research, they are still considered as the reference method for motion capture.

Consequently, other approaches based on alternative technologies have been investigated over the past years. For example, wearable Inertial Measurement Units (IMUs) offer the advantage of avoiding all camera-related issues such as field of view, occlusions, and controlled environment [9]. They still have the disadvantage of being an external equipment to still wear, involving high technical skills, and being sensitive to ferromagnetic disturbances and exposed to drift over time [10]. Another approach involves depth-field cameras (RGBD), which give access to a 2.5D world (only the depth of the front facing plane of view is measured), or even to full 3D with a network of few RGBD cameras [11,12,13]. On the other hand, these cameras suffer from a sensitivity to lightning conditions, they work at lower framerates, and they are short-range [14].

**Figure 1:**
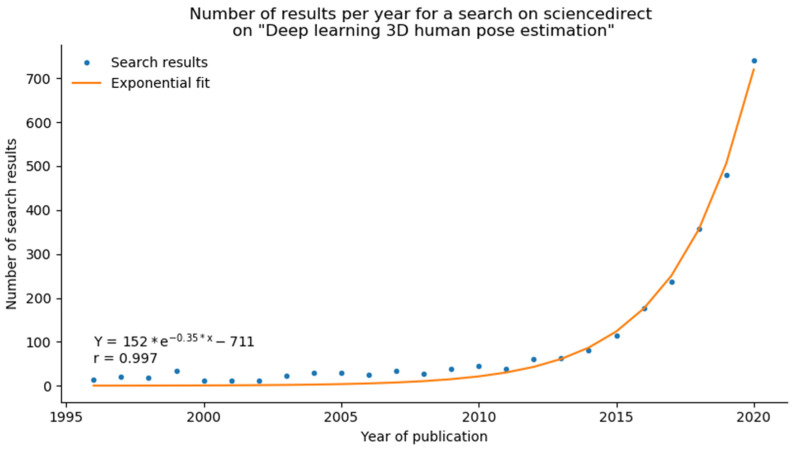
The search for “deep learning 3D human pose estimation” (dots) fits an exponential curve line. The search produced less than 100 results until 2015. Over the course of 5 years, the number has reached almost 750.

A recent breakthrough has come from Computer Vision. The explosion of deep-learning based methods from 2D camera videos, for which the research has skyrocketed around 2016 [15], is related to the increase in storage capacities and huge improvements in GPU computing. A search on ScienceDirect for “deep learning 3D human pose estimation” produced fewer than 100 papers until 2015, and the number has now reached almost 750 over the span of 5 years, fitting an exponential curve (Figure 1).

There has been rekindled interest from the Biomechanics community towards image-based kinematics, which is where it all started with the invention of chronophotograph in the 19th century by Marey in France, and Muybridge in the USA [16]. Currently, two approaches coexist in human and animal motion analysis for pose estimation: on the one hand, computer vision using deep-learning techniques mostly focus on joint positions only, while the interest in biomechanics lies in kinematics, which involves joint angles. One of the main challenges is to bridge the gap between these two worlds and to take advantage of deep-learning technologies for kinematic analysis [17,18].

### 1.2. 2D Pose Estimation

The most well-known, off-the-shelf 2D human pose estimation solutions are OpenPose [19] and, to a lesser extent, AlphaPose [20]. While both show similar results, OpenPose has the advantage of being a bottom-up approach, whose computation time does not increase with the number of persons detected [19]. A bottom-up approach first detects all available joint keypoints and then associates them to the right persons; a top–bottom approach first detects bounding boxes around each person and then finds joint keypoints inside of them. OpenPose has been trained on the CMU Panoptic Dataset [21], with 511 synchronized videos of multiple people in motion, alone or engaged in social activities. Another 2D pose estimation toolbox is DeepLabCut [22], which was initially intended for markerless animal pose estimation, but can be custom trained for the detection of any human or non-human keypoint with a relatively small dataset. All of these tools are open-source. Other approaches have shown even better results on evaluation datasets (see Chen et al. [23]), but they are generally slower and not as widespread.

### 1.3. 2D Kinematics from 2D Pose Estimation

Some authors have bridged 2D pose estimation to more biomechanically inspired goals, such as gait kinematics analysis. Kidzinski et al. present a toolbox for quantifying gait pathology that runs in a Google Colab [24]. Stenum et al. [25] evaluated gait kinematics calculated from OpenPose input concurrently with a marker-based method. Mean absolute error of hip, knee, and ankle sagittal angles were 4.0°, 5.6°, and 7.4°, respectively [25]. Liao et al. [26] have not released their code, but they used OpenPose outputs to train a model invariant to view. Viswakumar et al. [27] performed direct calculation of the knee angle from an average phone camera processed by OpenPose. They showed that OpenPose is robust to challenging clothing such as large Indian pants, as well as to extreme lightning conditions. Other sports activities have been investigated, such as lower body kinematics of vertical jumps [28] or underwater running [29]. Both works trained their own model with DeepLabCut. Serrancoli et al. [30] fused OpenPose and force sensors to retrieve joint dynamics in a pedaling task. 

### 1.4. 3D Pose Estimation

There are a lot of different approaches for markerless 3D human pose estimation and listing them all is beyond our scope (see Wang et al. [15]). Some older approaches are not based on deep learning and require specific lightning and background conditions, such as visual-hull reconstruction [31]. Some directly lift 3D from a single 2D camera (see Chen et al. [23]), with different purposes: one estimates the positions of a set of keypoints around the joint instead of determining only the joint center keypoint so that axial rotation along the limb is solved (see Fish and Clark [32]); SMPL and its sequels retrieve not only joint positions and orientations, but also body shape parameters (see Loper et al. [33]), while XNect primarily focuses on real time (see Mehta et al. [34]). A few approaches even strive to estimate 3D dynamics and contact forces from a 2D video input (see Rempe et al. [35], Li et al. [36]). Rempe et al. solved occlusions from a 2D input [37], but this remains a probabilistic guess that may be unsuccessful in case of unconventional positions of hidden limbs, whereas using more cameras would have given more trustworthy results. Haralabidis et al. fused OpenPose results from a single monocular video and two IMU outputs and solved kinematics of the upper body in OpenSim (an open-source biomechanical 3D analysis software [38,39]) in order to examine the effects of fatigue on boxing [40].

Some research attempts to solve 3D pose estimation from a network of uncalibrated cameras, i.e., cameras whose extrinsic parameters (translation and rotation with respect to the coordinate system), intrinsic parameters (focal length, pixel size, etc.), and distortion coefficients are not known. It either uses 2D pose estimations of each view as visual cues to calibrate on (see Takahashi et al. [41]), or an adversarial network that predicts views of other cameras, compares them to real views, and adjusts its calibration accordingly (see Ershadi-Nasab et al. [42]). Dong et al. [43] recovered 3D human motion from unsynchronized and uncalibrated videos of a repeatable movement found on internet videos (such as a tennis serve performed by a celebrity). Using uncalibrated videos is still a very experimental trend that requires more research before being used in biomechanics.

We chose to focus on the methods that estimate 3D pose by triangulating 2D pose estimations from a network of multiple calibrated cameras. The classical evaluation metric is the MPJPE (Mean Per Joint Position Error), which is the average Euclidian distance between the estimated and the ground truth joint coordinate. Most methods take OpenPose as an input for triangulation, and more specifically the body_25 model. Labuguen et al. evaluated 3D joint positions of a pop dancer with a simple Direct Linear Transform triangulation (DLT [44,45]) from four cameras [46]. Apart from the upper body for which error increases to almost 700 mm, the average joint position error is about 100 mm. Nakano et al. examined three motor tasks (walking, countermovement jumping, and ball throwing), captured with five cameras and triangulated with the same methods, with a subsequent Butterworth filter [47]. A total of 47% of the errors are under 20 mm, 80% under 30 mm, and 10% are above 40 mm. The largest errors are mostly caused by OpenPose wrongly tracking a joint, for example, by swapping the left and the right limb, which causes large errors up to 700 mm. This may be fixed either by using a better 2D pose estimator or by using more cameras to reduce the impact of an error on a camera, or else by considering the temporal continuity in movement.

Slembrouck at al. [48] went a step further and tackled the issue of limb swapping and multiple persons detection. In case of multiple persons detection, one needs to make sure they triangulate the person detected on one camera to the same person detected on the other ones. They managed to associate people across cameras by examining all the available triangulations for the neck and mid-hip joints: the people were the same when the distance between the triangulated point and the line defined by the detected point and the camera center was below a certain threshold. They only focused on lower limb. Their first trial featured a person running while being filmed by seven cameras, whereas their second one involves a person performing stationary movements such as squats while being filmed by three cameras. After filtering, the average positional error in the first case was about 40 mm, and it was roughly 30 mm in the second case (less than 20 mm for the ankle joint). Other authors dealt with the multi-person issue in a slightly different way [49,50,51]. In average, if the detected persons are correctly associated and the limbs do not swap, the average joint position error for an OpenPose triangulation is mostly below 40 mm.

Some triangulation methods not based on OpenPose achieved even better results on benchmarks, although this comes at the cost of either requiring heavy computations or of being out of reach for non-experts in computer vision. The classic approach is to detect probability maps for each joint, to assume that the maximum probability is the actual 2D joint position, and then to triangulating these scalar positions. Instead of this, the main two state-of-the art methods directly perform a volumetric triangulation of the heatmaps, and only then take the maximum probability. By working this way, they keep all the information available for as possible for as long as possible. They manage to lower their MPJPE to about 20 mm (See He et al. [52], Iskakov et al. [53]).

### 1.5. 3D Kinematics from 3D Pose Estimation

Instead of just working on 3D joint positions, the issue of 3D markerless kinematics (i.e., gait parameters and joint angles) is starting to be tackled. Zago et al. [54] evaluated gait parameters computed by triangulating two videos processed by OpenPose and noticed that straight gait direction, longer distance from subject to camera, and higher resolution made a big difference in accuracy. D’Antonio at al. [55] performed a simple triangulation of the OpenPose output of two cameras and computed direct Euler angle calculations for the lower limb. They compared their results to those of IMU and pointed out that errors were higher for running than for walking, and were also rather inconsistent (by up to 14°), although they decreased from 2° to 7° if the two cameras were set laterally rather than at the back of the subject. Theia3D, a recent commercial (and not open) solution, estimates the positions of a set of keypoints around the joint and then uses a multi-body optimization approach to solve inverse kinematics (See Kanko et al. [56,57]). They noticed an offset in hip and ankle angles between their markerless system and the reference marker-based one, likely due to different skeletal models. Once this offset was removed, the root mean square error (RMSE) in lower limb roughly ranged between 2° and 8° for flexion/extension and abduction/adduction angles, and up to 11.6° for internal/external rotation. AniPose has broadened the perspective to the kinematics of any human or animal with a DeepLabCut input, in comparison to OpenPose. They offer custom temporal filters as well as spatial constraints on limb lengths (see Karashchuck et al. [58]).

### 1.6. Robustness of Deep-Learning Approaches

According to the review of Desmarais et al. [59], the performance of a method can be ranked regarding its accuracy, speed, or robustness. Accuracy is mostly assessed with MPJPE; speed is evaluated either regarding computing complexity, or framerate when possible; and robustness is gauged through differences in the results while changing the system parameters only. Moeslund and Granum [60] proposed to express it as the number of constraints on the subject or on the environment required for a motion capture system to be operational.

Authors usually only consider accuracy (See Desmarais et al. [59]), although speed and robustness are of paramount importance in the context of sports, especially “in the wild”. Accuracy will be assessed in the next study (Part 2: Accuracy), and we do not address calculation speed here; only robustness will be considered.

Some of the assumptions proposed by Moeslund and Granum [60] have already been universally overcome by deep-learning-based methods. For example, no markers are involved anymore, the subject can wear their usual clothes (including loose pants or dresses [27]), and the background does not need to be static or uniform. Some other items remain an open problem.

For instance, most 3D methods assume that only one person lies in the camera field of view. This is a strong assumption, especially outdoors where people and athletes pass by and an operator is often present. Although it is starting to be addressed, this is still very debated [48,49,50,51].

Other open questions lie in the environment, which in a sports context is much less controlled than in a lab, which can result in bad image qualities. 2D kinematics methods usually assume a slow, continuous movement in the sagittal plane. This is rarely the case in sports. Viswakumar et al. [27] experienced that OpenPose was very robust to extreme lightning conditions. However, research has shown that pose estimations models are more robust to noise or brightness changes, while less robust to motion or to defocus blur (See Wang et al. [61]).

Occlusions are, for the most part, solved by using a network of calibrated cameras. Since triangulation is computed using a least square method, a large number of cameras will also blunt imprecisions on the 2D joint estimations. A recent study showed that once correctly trained for 3D macaque pose estimation, eight cameras were enough to correctly infer 80% of the 13 considered keypoints, while four cameras decreased the performance to about 50%. However, a correct estimation of extremities such as feet and hands required more than eight cameras (see Bala et al. [62]).

**Figure 2:**
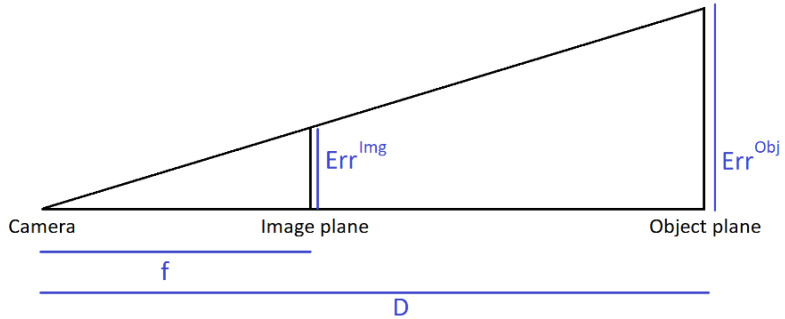
The pinhole camera model, permitting a correspondence between image coordinates and object coordinates. F—focal distance; D—object to camera distance; Err^Img^—error on image plane; Err^Obj^—error on object plane. F and Err^Img^ are usually expressed in pixels, while D and Err^Obj^ are expressed in meters.

Camera calibration can be challenging outside with bright light and contrasting shades and is close to impossible with the classic approach based on predefined objects with markers. Moreover, calibrating with a checkerboard may cause more errors on intrinsic and extrinsic camera parameters estimation (See Sun and Cooperstock [63]). A calibration is generally considered acceptable if the average residuals of each camera (i.e., the root mean square error of the reprojection of the 3D reconstructed coordinates on the 2D image plane) is below 1 px. In metric terms, the markers-based Qualisys Track Manager software recommends redoing a calibration when the average residuals exceed 3 mm [64]. The pinhole camera model (See Dawson-Howe and Vernon [65]) gives an equivalence between pixel values on the image plane and metric values on the object plane at the center of the scene, as demonstrated by Figure 2 and Equation (1).

(1) $${\mathrm{Err}}^{\mathrm{Img}}=\frac{{\mathrm{Err}}^{\mathrm{Obj}}\times \;f}{D},$$

### 1.7. Objectives of the Study

As shown above, attempts at producing thorough 3D markerless kinematics tools are still scarce. The aim of this article is two-fold. First, to introduce Pose2Sim (we plan to release the code as open source with Part 2 of this series of articles), a simple and free tool bridging OpenPose [19] to OpenSim [38,39], and second, to assess the robustness of the workflow.

The relevance of the computed 3D full-body angles is estimated by comparing our angle results to those of a normative walking database. Further concurrent validation of the accuracy will be determined in Part 2 of this series of articles. Repeatability is evaluated by comparing movement cycles to each other, within each task and each capture condition. Robustness itself is assessed through different movements, in accordance with the open problems previously described. First, in addition to the person of interest, some people are present in the background and thus detected. Additionally, the analysis is challenged regarding (1) image quality by simulating a dark scene captured with defocused cameras objectives, (2) occlusions by decreasing the number of cameras, and (3) calibration errors by corrupting calibration results. The underlying idea presented in this article is to show that modifying external environment parameters does not impact variabilities in joint angle estimation.

## 2. Materials and Methods

### 2.1. Data Collection

#### 2.1.1. Experimental Setup

To guarantee a tight control of the environment parameters, we captured our data in a dedicated studio platform, which is able to create realistic virtual views similar to outdoor video. This platform was a 10 m × 10 m × 5.6 m green room equipped with 68 cameras recording at 30 fps in 4 Mpixels resolution, for a practical acquisition space of about 5 m × 5 m × 3 m. The system computes 3D textured meshes by convex visual hull reconstruction (See Laurentini [66]). The meshes were inserted in a virtual environment composed of an environment texture map captured from a BMX racetrack, and a custom-made virtual floor. It should be noted that three people were present in the background, which introduced a realistic artefact of multiple subjects.

**Figure 3:**
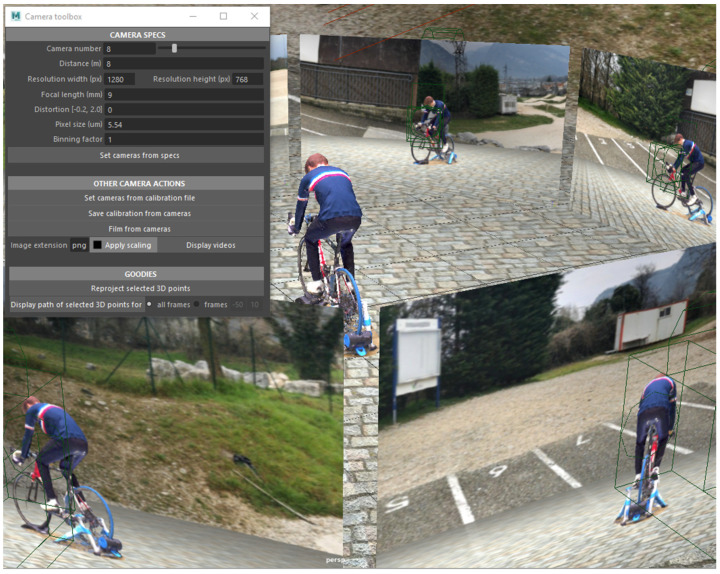
The camera toolbox developed for Autodesk Maya, which lets one set up virtual cameras, save their parameters in a calibration file, and film realistic looking synthetic videos. Here, we set up 8 video cameras, regularly distributed 8 m away from the subject.

We developed a script for the Autodesk Maya (similarly, we plan to release the code as open source with Part 2 of this series of articles) software that allows us to render the textured mesh files, as well as to virtually set any cameras with specific properties (position, orientation, resolution, focal length, distortion, pixel size, binning factor). Views seen through virtual cameras can be saved as video files and visualized into a global 3D environment (Figure 3). The generated video files were used as input to the 3D kinematics pipeline.

**Figure 4:**
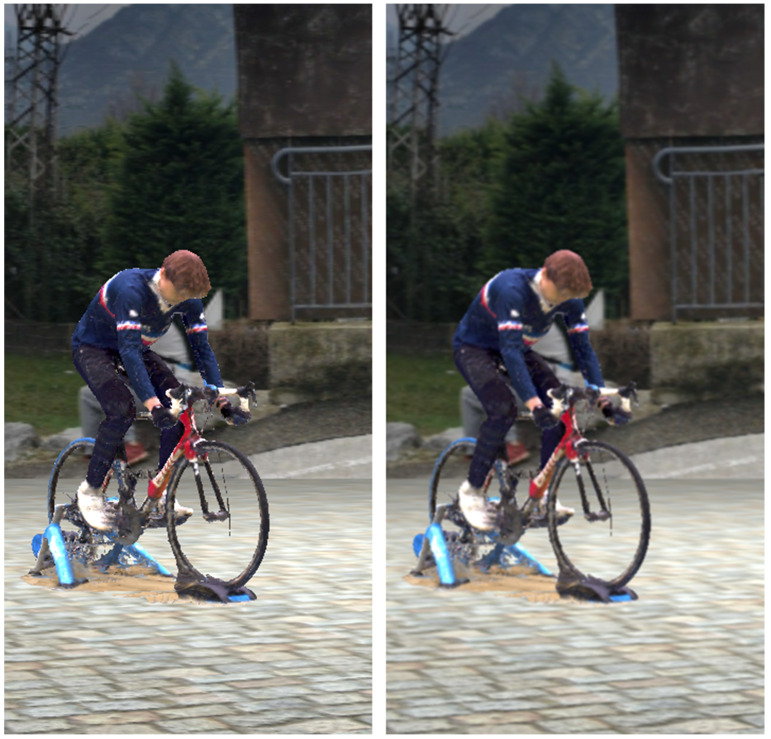
To smooth out sharp edges due to compositing, we applied a 3 × 3 pixel Gaussian blur to the videos filmed from our virtual scene.

For the purpose of this study, we created 8 virtual video cameras (resolution 1280 × 768 px, focal length 9 mm, no distortion, pixel size 5.54 µm, and no binning). Binning refers to the process of grouping pixels in order to increase sensitivity to light at the expense of decreasing resolution. Cameras were regularly distributed 8 m away from the center of the captured volume, at a height of 1 m, so that the whole body could be detected for a maximum of cycles. We then rendered the scene as video files from our virtual cameras and saved the exact calibration parameters. We applied a 3 × 3 pixel Gaussian blur afterwards to reduce sharp edges of the virtual scene compositing (Figure 4). This resulting image quality is considered as “standard”.

#### 2.1.2. Participant and Protocol

One healthy adult male subject (1.89 m, 69 kg) participated in the study. He provided his informed written consent prior to participating. The study was conducted in accordance with the Declaration of Helsinki. No requirement was given to him regarding his outfit. He was asked to perform three basic sports tasks: walking, running, and cycling. For all of the tasks, the subject was given a moment beforehand to warm up and find a comfortable and regular pace, which he could then follow owing to the sound of a metronome:

1. Walking: The subject walked in a straight line back and forth over the 10 m diagonal of the room. His body mesh could be fully reconstructed only in the central 5 m of the acquisition space, i.e., only roughly 2 gait cycles were acquired per walking line. His comfortable stride pace was 100 BPM (Beats per Minute). The stride length was not monitored.
2. Running: The subject jogged in a straight line back and forth along the 10m diagonal of the room. His comfortable stride pace was 150 BPM (Beats per Minute). The stride length was not monitored.
3. Cycling: The subject cycled on a road bike placed on a home trainer. He himself adjusted the resistance and the height of the saddle prior to the capture. His comfortable cadence was 60 BPM.

As obtaining the textured meshes of the subject in the green Kinovis room involved filming simultaneously with 68 4Mpixels cameras that generated a flow of over 8 gigabytes per second, the capture design limited the acquisition time to 45 s.

### 2.2. The Reference Condition and the Three Degraded

#### 2.2.1. Reference Condition (Ref)

The reference condition under which our 3D markerless kinematic system had to operate took advantage of the standard image quality, 8 available virtual cameras, and a perfect calibration. The standard quality was the best we could use. The reference condition involved 8 virtual cameras as a good compromise of what is feasible in real outdoor conditions. Moreover, a study on macaques showed that 8 cameras were enough to correctly infer 80% of the 13 considered keypoints (see Bala et al. [62]). The calibration could be considered perfect, since the virtual cameras were explicitly specified in the virtual environment.

#### 2.2.2. Poor Image Quality (Im)

**Figure 5:**
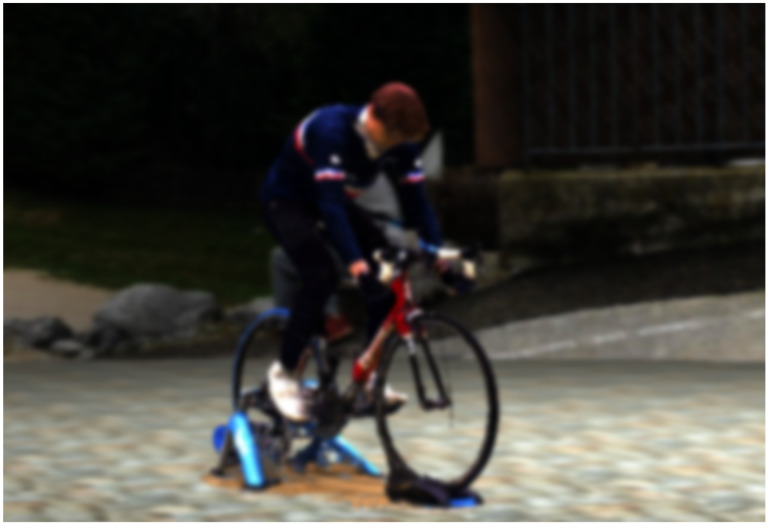
The image under poor image quality (Im) conditions. A Gaussian blur (11 × 11 px) was applied, and a 0.5 gamma compression made the image darker.

Video quality was made blurrier and darker: a Gaussian blur (11 × 11 px) was applied, as well as a 0.5 gamma compression (Figure 5). This simulated a dark scene captured with defocused camera objectives.

#### 2.2.3. Less Cameras (4c)

The 2D joint coordinates were triangulated with only 4 cameras, instead of 8 in the reference condition: one on each side, one in the front, and one in the back, set 90° apart from each other. 

#### 2.2.4. Calibration Errors (Cal)

Calibration residuals are classically supposed to be under 1 px on the image plane or under 3 mm on the object plane. Using Equation (1) demonstrates that in our case 3 mm corresponds to 0.61 px. We chose to simulate a calibration error of 2 px, which corresponds to about 1 cm (Equation (2)).

(2) $${\mathrm{Err}}^{\mathrm{Obj}}=\frac{{\mathrm{Err}}^{\mathrm{Img}}\times \;D}{f}=\frac{2\times 8}{\frac{9\times 10^{-3}}{5.54\times 10^{-6}}}=9.8\times 10^{-3}\;m$$

The calibration error was simulated by translating the extrinsic parameters of each camera in a random direction. The norm was randomly picked in a normal distribution of mean 2 px and a standard deviation of 1 px. The mean of these 8 translations was ensured to be equal to 2 ± 10^−3^ px.

### 2.3. OpenPose: 2D Pose Estimation

**Figure 6:**
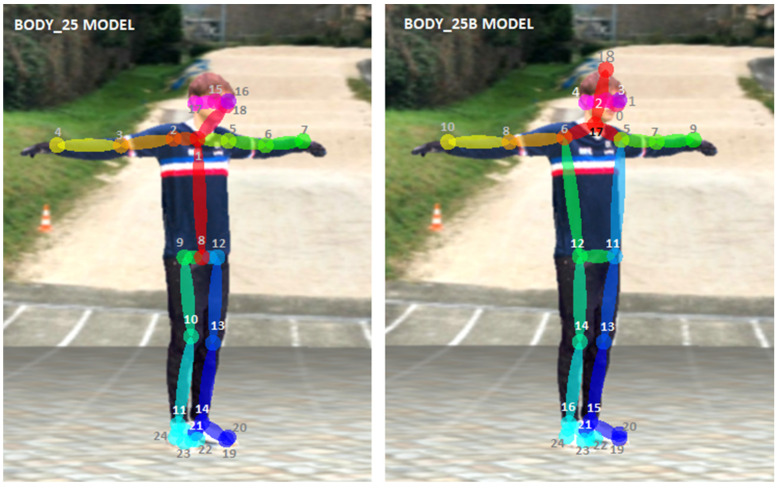
The experimental body_25b OpenPose model is more accurate than the default body_25 one. As an example, the left knee is slightly misplaced on the default model. The keypoint definition differs between both models.

We applied OpenPose (version 1.6) on all the captured videos. We used the experimental body_25b model [67] with highest accuracy parameters, which is more accurate than the default body_25 one and reduces the number of false positives. Its keypoint definition differs slightly: it adds the MPII head and neck keypoints and removes the artificially created neck and middle hip of the body_25 model (which are simply the middle point of the shoulders and the hips), see Figure 6.

### 2.4. Pose2Sim: 3D Pose Estimation Toolbox

Pose2Sim robustly triangulates OpenPose outputs from several calibrated cameras and feeds the resulting 3D joint coordinates to OpenSim. The exact same parameters were used for all 4 conditions and all 3 movement tasks in order to make sure the process did not induce any supplementary deviation to the compared results.

#### 2.4.1. Tracking the Person of Interest

One needs to differentiate the people in the background from the actual subject. The tracking step examined all possible triangulations of a chosen keypoint (e.g., the neck) among all detected persons and reprojected them on the image planes. The triangulation with the smallest reprojection error was the one associating the right person on all cameras. If the reprojection error was above a certain threshold, the process was repeated after taking off one, or several, cameras. This happened, for example, if the person of interest had exited the field of view of a camera while another person was still in its background; subsequently, this camera had to be taken off.

We chose to triangulate the neck keypoint, and the reprojection error threshold was set to 10 px.

#### 2.4.2. Triangulation

The classic direct linear transformation (DLT) triangulation (see Hartley and Sturn [44]) (Algorithm 1) was enhanced by weighting it with the confidence OpenPose gives to each of its keypoint estimations (Algorithm 2). This is much faster than a volumetric triangulation of heatmaps (see Iskakov [53]), but it still takes advantage of some confidence information.

Some keypoints were sometimes occluded to some cameras, either by the subject himself, by his cycling gear, or simply because the subject left the camera field of view. In such a case, OpenPose usually gave a low (or zero) confidence to the estimated point, which was dealt with by setting a confidence threshold above which the camera in question was not used for the triangulation. However, OpenPose occasionally wrongly detected the occluded keypoint with a relatively high confidence. Under such circumstances, the point was erroneously triangulated. This issue was spotted and solved by reprojecting the 3D point on the camera planes. If the reprojection error between the reprojected points and the OpenPose detection was higher than a predefined threshold, the process was repeated after removing one, or several, cameras. If less than 3 cameras remained, the frame was dropped for this point, and missing frames were later interpolated with a cubic spline. 3D joints positions were then exported as an OpenSim compatible .trc file.

We chose a confidence threshold of 0.3 and a reprojection error threshold of 10 px.

**Algorithm** **1.** *Proof of the classic direct linear transformation (DLT), commonly used to solve the triangulation of 2D coordinates from several cameras with a least square approach*.

Let **Q** = (X, Y, Z, 1) be the homogeneous coordinates of a 3D object point, **q** = (u, v, 1) the homogeneous coordinates of a 2D image point on a given camera, **P** = (P_1_^T^, P_2_^T^, P_3_^T^, P_4_^T^) the projection matrix of the same camera, with P_1_^T^, P_2_^T^, P_3_^T^, P_4_^T^ the rows of **P**, and **λ** an unknown scale factor. The equation

**λq** = **P**
**Q**, (3)

may be written as

**λ**u = P_1_^T^**Q**, **λ**v = P_2_^T^**Q**, **λ** = P_3_^T^
**Q**, (4)

which gives two equations:

(P_1_^T^ − u P_3_^T^) **Q** = 0, (P_2_^T^ − v P_3_^T^) **Q** = 0. (5)

With N cameras, we obtain a system of 2N equations that can be written in the form:

A **Q** = 0. (6)

A singular value decomposition (SVD) of A leads to a least square solution for our 3D object point **Q**. Indeed, A can be expressed as A = USV^T^ with U, V orthonormal bases, and S the diagonal matrix of the singular values (σ_1_, σ_2_, σ_3_, σ_4_) of A. **Q** can be expressed as **Q** = V α, with α = (α_1_, α_2_, α_3_, α_4_). Now, minimizing (A**Q**)² also minimizes A**Q**:

(7) $$\begin{array}{l} {\left(A\;Q\right)}^{2}={\left(A\;Q\right)}^{T}\;\left(A\;Q\right)\\ =\left(\alpha^{T}V^{T}{\;\mathrm{VSU}}^{T}\right)\left({\mathrm{USV}}^{T}\;V\alpha \right)\\ =\alpha^{T}\;S\;\alpha \\ =\sum_{i=1}^{4}\alpha_{i}^{2}\sigma_{i}^{2} \end{array}$$

which is minimum when all but the smallest singular value are set to zero. For example, if σ_min_ = σ_4_, then A**Q_min_** = α_4_ σ_4_. Then, **Q** = V_4_ α_4_ = (X, Y, Z, 1). As a consequence, the coordinates of the triangulated point are

X = V_14_/V_44,_ Y = V_24_/V_44,_ Z = V_34_/V_44_. (8)

**Algorithm** **2** . *Our weighted direct linear transformation (DLT), which takes OpenPose’s confidence into account*.

Our weighted DLT simply consists of weighing the equations in Equation (5) with the confidence **c** that OpenPose gives for each camera. This leads to Equation (9). The rest of the procedure remains unchanged.

(9) $$c\times \left(P_{1}^{T}-{u\;P}_{3}^{T}\right)\;Q=0,\;c\times \left(P_{2}^{T}-{v\;P}_{3}^{T}\right)\;Q=0.\;$$

#### 2.4.3. Filtering

3D coordinates are filtered with a Butterworth filter. We chose a zero-lag fourth order low-pass Butterworth filter, with a 6 Hz cutoff frequency.

### 2.5. OpenSim: Joint Angle Calculations

We used the software OpenSim (version 4.2) for biomechanically consistent inverse kinematics.

#### 2.5.1. Gait Events

Cycles were determined using one of the methods given by Zeni et al. [68] for the determination of gait events using kinematic data. A cycle was defined as starting from heel strike, with a duration t_cycle_:

(10) $$t_{\mathrm{cycle}}=\frac{2}{\mathrm{cadence}/60}\;\mathrm{seconds}.\;$$

Zeni et al. [68] suggested defining heel strike as the time of maximum distance between the heel and the sacrum. Here, the sacrum marker was assimilated to the ipsilateral hip joint coordinate. Although there is no heel strike in cycling, we used the same definition as a reference for the start of a cycle. An implementation of the algorithm is given in the Utils section of Pose2Sim. The duration of a cycle t_cycle_ was 1.2 s for walking, 0.8 s for running, and 1.0 s for cycling. The reduced area of acquisition and the limit of 45 s of capture restricted the analysis to 8, 9, and 15 cycles for walking, running, and cycling, respectively.

#### 2.5.2. Model Definition

The full-body musculo-skeletal model proposed by Rajagopal et al. [69] was adjusted to our needs. The proposed markerset was replaced with the OpenPose one. Markers were carefully placed on the body as closely as possible to their 2D pose correspondences. Muscles were removed since they were not considered at that stage. A ball joint was added between head and torso so that the rotation of the head could be roughly rendered. Pelvis translation and subtalar angle rotation were unlocked. Lumbar extension and lumbar bending were clamped between −15° and 15°, and constraints on hip flexion/extension ranges of motion were released since they were too strict for our cycling task. This model uses a coupling relationship between knee flexion and the other planes of movement, which allows for 3D knee kinematics with only one degree of freedom.

#### 2.5.3. Model Scaling

Scaling in OpenSim can be broken down into two parts: first, the proper scaling of the model, which adjusts bone dimensions and inertial properties according to the (independently calculated) virtual joint markers positions; second, the adjustment of the other markers on the model, especially anatomical and cluster markers. If the markerless model is defined properly, there is no need for further marker adjustments since they will not be subject to placement variation due to human error or to skin movement artifact.

Bones were scaled so that model markers q_m_ matched the experimental markers q_e_. Each body was scaled according to a factor computed as a ratio of the distance between the corresponding q_m_ and q_e_. The markers used for scaling bodies were chosen as follows:

- Arm: pairs (left shoulder, left elbow) and (right shoulder, right elbow);
- Forearm: pairs (left elbow, left wrist) and (right elbow, right wrist);
- Thigh: pairs (left hip, left knee) and (right hip, right knee);
- Shank: pairs (left knee, left ankle) and (right knee, right ankle);
- Foot: pairs (left heel, left big toe) and (right heel, right big toe);
- Pelvis: pair (right hip, left hip);
- Torso: pairs (neck, right hip) and (neck, left hip);
- Head: pairs (head, nose);

All weights of joint coordinates were set to 1, apart from those of the nose and the head, which were set to 0.2, and from those of the other head markers, which were set to 0. Since we processed scaling during a T-pose, we added as a condition that the ankle flexions should be fixed at a neutral 0° angle.

#### 2.5.4. Inverse Kinematics

**Figure 7:**
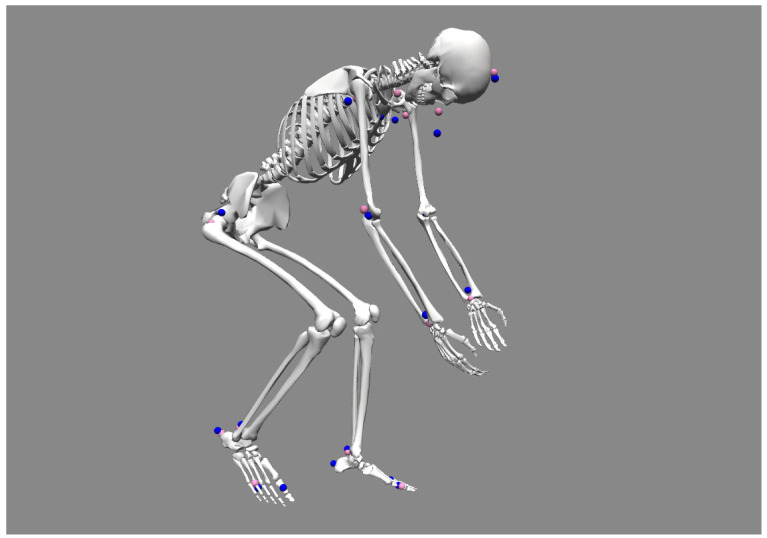
OpenSim inverse kinematics on cycling (C). Model markers are pink; experimental markers are blue.

The inverse kinematic tool was used with the same marker weights as in the scaling step (Figure 7). We visually verified that model markers approximately overlayed experimental markers, and we batched inverse kinematics for all gait cycles with the command line utilities. We then concatenated all results in a .csv file analyzed them using python.

### 2.6. Statistical Analysis

As similar results were expected for both sides, as more strides were captured on the left one, and for the sake of clarity, the analysis was performed on the left side only. Descriptive statistics were calculated at the triangulation stage, for each (Ref), (Im), (4c), and (Cal) condition. We calculated mean, standard deviation (std), and maximum (max) number of cameras excluded for performing an acceptable triangulation (i.e., OpenPose confidence above 0.3 and reprojection error below 10 px.), as well as mean, std, and max reprojection errors. Descriptive statistics of the OpenSim scaling and inverse kinematics steps were also rendered: we calculated mean, std, and max root mean square error (RMSE) of the experimental and model markers.

Next, we investigated the relevance of our results. The angles of the Ref condition on the walking task were compared to those of a normative walking gait database (See Fukuchi et al. [70]), from which we took a subset of 14 young, healthy, males walking at a “comfortable” speed (Norm). Pearson’s correlations (r) and mean absolute error (MAE) were calculated. The angle definition was different from that in the OpenSim model (See Trinler et al. [71]); only the flexion/extension angles were compared.

Repeatability was estimated by computing stride-to-stride standard deviations within (Ref), (Im), (4c), and (Cal) conditions. The results were compared to Kang and Dingwell’s [72], obtained with a marker-based approach and averaged over 18 young adults. Th robustness of the workflow was assessed by calculating the standard deviation ratio between each degraded condition and the Ref one, as well as the r coefficient and the MAE. Statistical parametric analysis (SPM-1D) was lastly performed between Ref and degraded conditions to convey statistical differences along time (See Warmenhoven [73]).

## 3. Results

### 3.1. Data Collection and 2D Pose Estimation

Each trial took several hours to process on a cluster of high-end computation units. Then, filming the resulting 3D meshes in a virtual scene with virtual cameras also took a few hours per sequence, as well as about 50 Go of storage space. Although all of this allowed us to perfectly control the parameters of the capture, this step would not be carried out in the wild, where capture would simply be carried out by filming with calibrated cameras.

Apart from the capture design, which is very particular to this study, the Openpose 2D pose estimation was the most computationally costly step. On a standard computer with an Nvidia GeForce GTX 1080 graphic card (8 Go memory), the detection for each camera ran at a little over 0.5 fps (i.e., 0.07 fps for 8 cameras).

### 3.2. Pose2Sim Tracking, Triangulation, and Filtering

On our standard computer, tracking was performed at an average of 5 fps depending on the number of undesired persons present in the background; triangulation was at about 10 fps; and filtering was almost instantaneous in comparison.

Depending on the considered frame, the reprojection error of a joint coordinate sometimes exceeded the prescribed threshold. In this case, the triangulation process was executed another time after excluding the 2D joint coordinates estimated from one, or several, cameras. Over all frames, an average of approximately 0.5 cameras were excluded in walking and in running and 1.6 in cycling (Table 1). The mean of the reprojection error was about 3.5 px (~1.6 cm) in walking and running and 6 px (~3 cm) in cycling. More cameras had to be excluded for the nose and for the extremities such as wrists and toes, and the reprojection error was mostly large on hips, head, and extremities.

Within each task, almost twice as many cameras had to be excluded in the (Im) condition as in the (Ref) condition, with nearly one camera excluded in walking and running, and nearly 2.5 in cycling. However, the reprojection error was increased by only about 10%. About twice as few cameras had to be excluded in the (4c) condition as in (Ref) condition, and the reprojection error was decreased by about 10%. The (Cal) condition did not involve more excluded cameras; indeed, calibration errors had no effect on the OpenPose confidence, and reprojection error stayed within the threshold of 10 px. However, a 2 px average error in calibration logically resulted in about a 2 px (~1 cm) larger reprojection error than in the Ref condition.

### 3.3. OpenSim Scaling and Inverse Kinematics

Scaling took a few hours, which is in line with the usual expectations in marker-based methods. However, markers would be positioned in the same place by the pose estimation algorithm regardless of the subject and of the operator: as a consequence, in the future, only bones would have to be scaled, and markers will not have to be further adjusted. Due to the small number of markers whose positions had to be optimized, Opensim’s inverse kinematics ran at more than 0.5 fps.

OpenSim recommends the RMS of experimental vs. model marker errors to be below 1 cm for scaling. Our best average RMS error was 1.9 cm. During inverse kinematics, it is recommended for it to stay below 2–4 cm. The average RMS error was typically below 4 cm, but it could reach much more for some markers at certain phases of the cycle, especially in the cycling task. Within each task, changing conditions made very little difference in RMS marker errors, including in mean, standard deviation (std), or maximum error (Table 2).

One should not compare the values of the reprojection errors in the triangulation step and of the marker errors in the inverse kinematics one. Primarily, the first one is calculated over time, while the second one is calculated over markers. Additionally, the first one is a mean absolute error (MAE), while the second one is a root mean square error (RMSE). RMSE squares the errors, which always makes it larger than the MAE. It penalizes large errors more, which has some implications that are not addressed here but are documented in Chai and Draxler [74]. These errors should only be used to compare conditions within each step.

### 3.4. Relevance, Repeatability and Robustness of Angles Results

**Figure 8:**
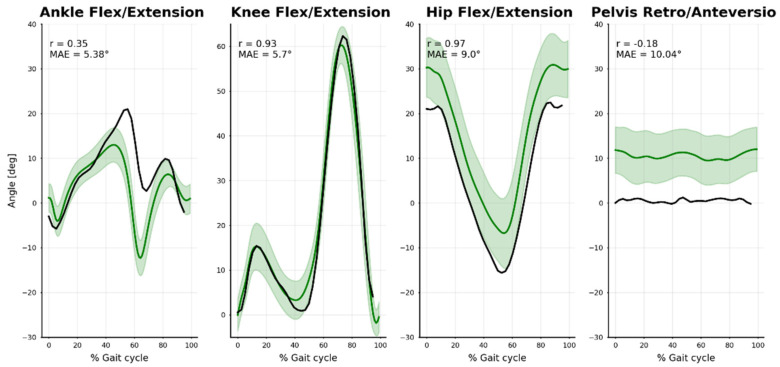
Comparison between our markerless results (black line: mean stride-to-stride results for our subject) and the normative marker-based database (green line and area: mean and standard deviation across 14 young, healthy, male subjects). Mean absolute error (MAE) and Pearson correlation coefficient (r) are represented.

Flexion/extension lower-limb angles were compared to a normative walking gait database (See Trinler et al. [70]) (Figure 8). Ankle movement differed noticeably (r = 0.35, MAE = 5.4°), especially between 40% and 70% of the gait cycle. There was a good agreement for the knee (r = 0.93, MAE = 5.7°) and hip angles (r = 0.97, MAE = 9.0°) despite some notable offset in hip angle. A similar shift occurred in the pelvis ante/retroversion angle (not further analyzed).

When averaged over all joint angles, the stride-to-stride standard deviations lay between 1.7° and 3.2° for all conditions and tasks (Table 3). The walking task was the most variable, while the cycling one was the least variable; however, in the latter the upper body was static and artificially drew the mean down, which was revealed after removing it from the statistics (Table 3). In the walking task, the stride-to-stride standard deviation of our individual lower body angles was higher than Kang and Dingwell’s [72], who used a marker-based approach (Table 4).

**Figure 9:**
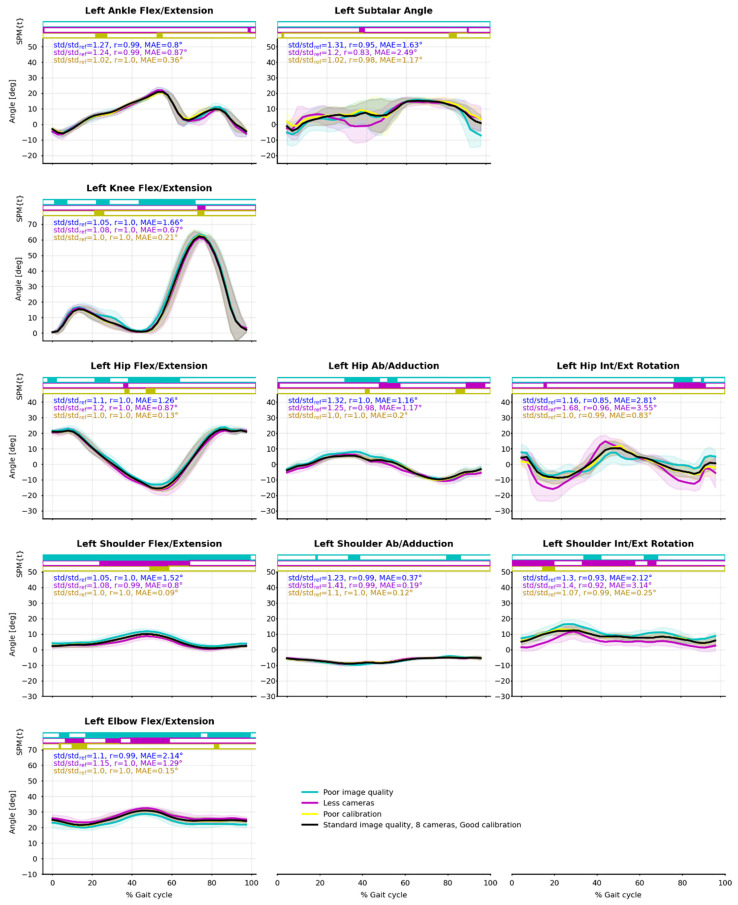
Joint angle means (solid line) and standard deviations (shaded area) from the eight captured cycles of walking. Reference condition (Ref) is black; degraded image quality (Im) is blue; four cameras instead of eight (4c) is purple; degraded calibration (Cal) is yellow. Pearson’s correlation coefficient (r) and mean absolute error (MAE) between Ref and Im, 4c, Cal are reported. Paired t-tests along time were computed by SPM-1D and are represented as bar plots above the curves: a color rectangle means that there was a cluster of statistically significant differences (α = 0.05) at that moment. Running and cycling figures can be found in the Supplementary Material.

**Figure A1:**
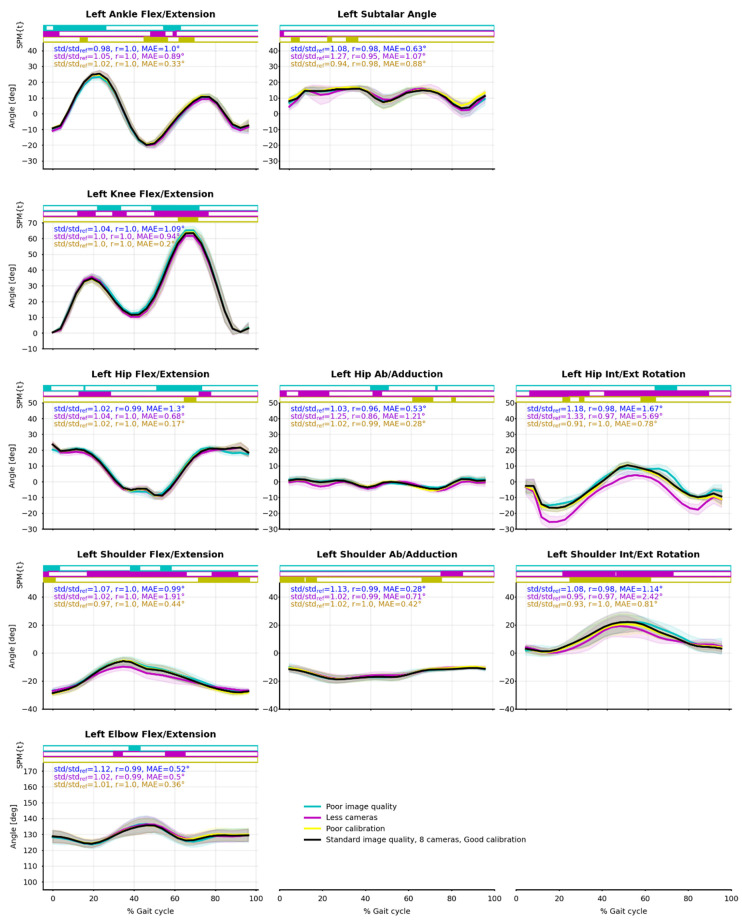
Joint angle means (solid line) and standard deviations (shaded area) from the nine captured cycles of running. Reference condition (Ref) is black; degraded image quality (Im) is blue; four cameras instead of eight (4c) is purple; degraded calibration (Cal) is yellow. Pearson’s correlation coefficient (r) and mean absolute error (MAE) between Ref and Im, 4c, Cal were calculated. Paired t-tests along time were computed by SPM-1D and are represented as bar plots above the curves: a color rectangle means that there was a cluster of statistically significant differences (α = 0.05) at that moment.

**Figure A2:**
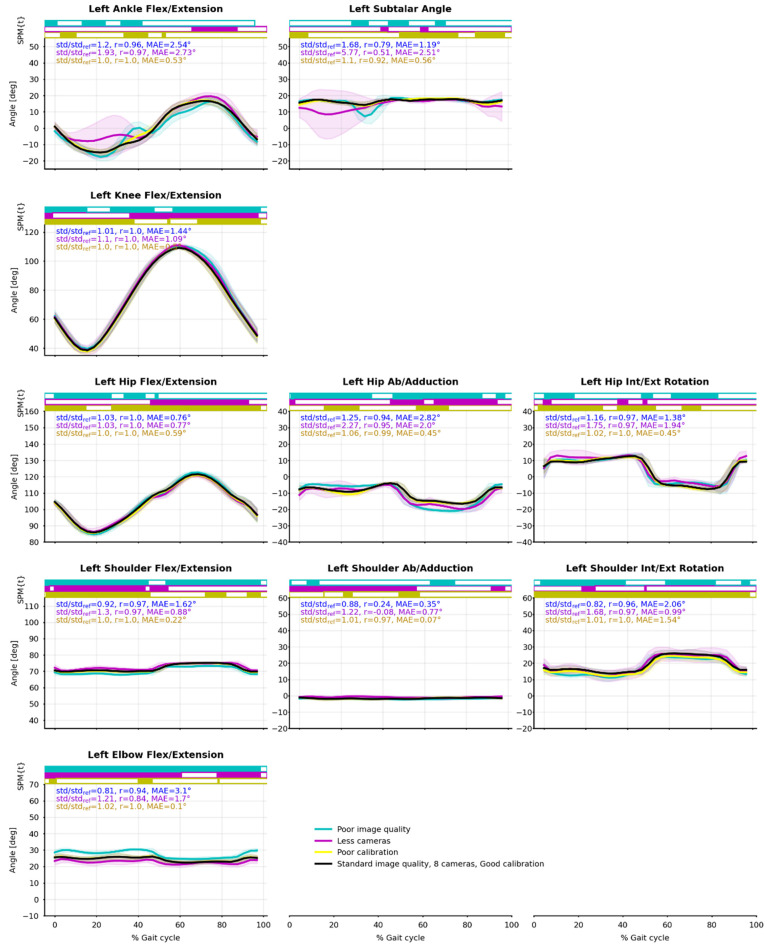
Joint angle means (solid line) and standard deviations (shaded area) from the 15 captured cycles of cycling. Reference condition (Ref) is black; degraded image quality (Im) is blue; four cameras instead of eight (4c) is purple; degraded calibration (Cal) is yellow. Pearson’s correlation coefficient (r) and mean absolute error (MAE) between Ref and Im, 4c, Cal were calculated. Paired t-tests along time were computed by SPM-1D and are represented as bar plots above the curves: a color rectangle means that there was a cluster of statistically significant differences (α = 0.05) at that moment.

There was a good agreement between the Ref condition and the degraded ones, at the same time across all tasks, movement planes, and joints. Mean absolute errors (MAE) averaged over all joint angles (compared to the reference condition—Ref) ranged between 0.35° and 1.7° for all conditions and tasks (Table 3). Individual angle MAE lay between 0.07° and 5.2° (Figure 9, Appendix A and Appendix B Figure A1 and Figure A2). However, the cycling task was more challenging: ankle joint angles were especially less robust regarding all considered dependent variables.

Angle results were particularly little affected by the 1 cm calibration error (Cal condition). The standard deviation between cycles (std) virtually did not increase, the average Pearson’s r correlation coefficient was 1.00 in all tasks but the cycling one (r = 0.98), and the mean absolute angle error stayed below 0.5° (Table 3).

The standard deviation in Im and 4c conditions increased by about 10 to 30% as compared to Ref condition, apart from the cycling 4c condition where it increased by 100%. The Pearson’s r correlation coefficient was about 0.98 in all tasks but the cycling one. Mean absolute angle errors across all joints lay between 0.9 and 1.8° (Table 3).

Kinematics were more robust in the flexion/extension plane (Table 5), where std generally did not increase by more than 10% as compared to the Ref condition, and r was mostly equal to 1.00. The difference in robustness was not as obvious in the MAE variable since the range of motion was much smaller in other planes.

The SPM showed some statistical differences along time between the reference condition and the degraded ones; however, they did not happen at any particular moment during the gait or pedaling cycle (Figure 9, Appendix A and Appendix B Figure A1 and Figure A2).

## 4. Discussion

### 4.1. Pose2Sim

Pose2Sim allows for seamless markerless kinematics from a network of calibrated and synchronized cameras by connecting two widespread, renowned, and open-source tools: the OpenPose 2D pose estimator and the OpenSim movement analysis software. It requires no marker placement, no specific cameras, and the scaling step is simpler than it is with markers-based methods. Pose2Sim integrates a tool for calibration, which can be performed either with a checkerboard (See Zhang [75]) or by converting a calibration file obtained with a marker-based method. Overall, human intervention is scarce, which makes it more robust to human error. 

Pose2Sim automatically tracks the person of interest when people are in the background, considers the confidence given by the OpenPose estimation, and is robust to missing frames and to the person exiting the field of view of one or several cameras. To the best of our knowledge, it is also the only one taking advantage of a solid anatomical 3D model in order to solve some rotations around the limb and to account for physical consistency. It is very customizable, in the sense that the user can adjust most parameters and even use a different 2D pose estimator if needed, including for non-human subjects (such as DeepLabCut for example.)

### 4.2. Relevance, Repeatibility, and Robustness

The walking angle results seemed to be relevant when compared to the normative database. The discrepancies may be due to a difference between the angle definition of both models, especially in the ankle: the normative database calculated direct Euler angles between limb segments, while our OpenSim model took bone geometries into consideration. The shift in the hip angle was related to an excessive pelvis anteversion, which may have been induced by the model being underconstrained due to our dearth of keypoints. This lack of marker constraints could be partly compensated by adding joint constraints, especially around the spine (See Beaucage-Gauvreau et al. [76]). In any case, the angle results seem to be relevant, but a further study will be needed to investigate their proper accuracy, as compared to a reference method exploiting a similar model definition.

Repeatability was simply assessed by the standard deviation of angle time series across gait cycles, although it also includes the participant’s intrinsic variability. However, the subject being young and healthy, his gait and cycling patterns were thought to be consistent. Hence, the results were repeatable as the standard deviation of angles across all strides within all tasks and conditions mostly stayed below 3°. Neveretheless, this standard deviation was still higher than the one previously reported by Kang and Dingwell for a young and healthy participant using a marker-based approach [72]. It may be partly caused by our lack of a force plate and our low sampling rate, which did not let us measure a very accurate heel strike event, and thus could have induced some additional variability. In addition, our videos were sampled at 30 Hz instead of hundreds of Hertz as is usual in motion analysis. It is to be noted that high-speed video cameras can sample at a such speed and solve at least a part of this issue. Depending on the sports task investigated, a 3° standard deviation can be more or less of an issue.

Robustness was investigated based on the criteria exposed by Moeslund and Granum [60]. First, people could be in the background. Then, several movement tasks have been investigated. Last, the workflow was confronted to three degraded conditions regarding image quality, camera number, and calibration. The results were also robust, since degraded conditions still gave consistent joint angles. A further SPM study showed occasional statistical differences along time, but did not reveal any part of the movement cycle to be more problematic than another. There was no apparent outlier in the 3D angle results, even in the degraded conditions. This was in spite of the presence of other people in the field of view and in spite of the subject walking out of it.

All stages of the workflow contributed to its robustness. First, we never had to deal with limb swapping, unlike other studies [47,48]. This may be due to our use of the experimental Body_25b OpenPose model, which is claimed to decrease the number of false positives [67]. Then, we used Pose2Sim to robustly sort the detected people and to triangulate 2D coordinates. These coordinates were the least precise in the Im condition where the image was strongly blurred and darkened, but Pose2Sim partly solved it by taking the OpenPose likelihood into account and by excluding the most inaccurate cameras: twice as many cameras were excluded as compared to the reference condition, which resulted in a reprojection error only 10% higher.

Finally, we used OpenSim to take advantage of a physically consistent full-body model and of an inverse kinematics optimization algorithm, which allowed us to refine our 3D data coordinates and to obtain 3D joint angles. The Cal condition simulated calibration errors, which bypassed the Pose2Sim safeguards since the OpenPose likelihood was unaffected, and the reprojection error stayed below the threshold. Despite it producing the largest reprojection error, virtually no difference with the reference condition was observed after the OpenSim pass. A 2 px calibration error (~1 cm) is much worse than the maximum 1 px, or 3 mm usually recommended, but the mean absolute error it induced for us stayed below 0.5°.

Using four cameras rather than eight still gave relevant angle results, but the individual errors of each camera cannot be blunted by the multiplicity of views, especially when faced with occlusion issues such as in the cycling task. The ankle joint angles were especially less robust regarding all considered dependent variables because the feet were more easily occluded by the cycling gear.

Ultimately, all conditions challenged the workflow at different stages, but the results remained stable and very close to those of the reference condition, with an average mean absolute error mostly lower than 1.5°, a correlation coefficient largely above 0.95, and a standard deviation usually increased by less than 20%. This demonstrates the robustness of the presented method in conditions that would have probably caused marker-based approaches to fail. Moreover, our reported angle deviations seem quite reasonable compared to errors in marker-based approaches, which can propagate up to 10° because of inter-operator differences (See della Croce et al. [4], Gorton et al. [5]), to 3° because of tissue artifacts (see Benoit et al. [6], Capozzo et al. [7]), or to 3° depending on the joint center calculation method (see Leboeuf et al. [8]). Essentially, the findings presented here seem to indicate that the slight decline in repeatability is an acceptable compromise when put into perspective with the increase in robustness, in ease of use, and in new opportunities for analysis of sports performed “in-the-wild”.

Nonetheless, it would be interesting to check for even more challenging conditions. We have investigated global Gaussian blur, but not movement blur, such as when the camera shutter speed is not high enough. It would also be interesting to investigate the effect of even larger calibration errors, even less cameras used, different camera placements, different camera types (such as GoPros with strong fisheye distortions), or different framerates or image definitions.

### 4.3. Limitations and Perspectives

Our use of a virtual scene helped us perfectly control all parameters of the capture. Although this scene is synthetically crafted, it looks similar to a real one. This is not thought to hinder the performance of 2D pose detection since deep-learning models are occasionally trained on synthetic datasets that look empirically less real and are still successful at augmenting ground-truth datasets (see Varol et al. [77]). However, an image with a better definition and with a framerate over 30 fps may improve results. Furthermore, this capture design limited us to a low level of data, with only one subject and about 10 cycles analyzed per task.

We noticed that the cycling task was particularly challenging, probably because of self-occlusions and gear occlusions. With marker-based methods, setting some cameras closer to the ankles and in a lower position would have slightly improved this issue. However, it was not an option here since OpenPose only works very well if the whole body is visible. Unlike D’Antonio [55], we did not find running to be more challenging for our system than walking. In the context of sports sciences, it would be useful to test other tasks, such as throwing (a typical 3D movement), flipping (the 2D pose estimator may have trouble with upside-down people), swimming (in an environment with a different refractive index, with splashes around limb extremities), or a sport discipline with unusual outfits and large occlusions (such as motorbiking or fencing.)

Moreover, and despite the system not being altered by people entering the field of view, we can currently only analyze the movement of one single person. For races, team sports, and combat sports, it would be useful to be able to analyze the movement of several athletes at the same time. This could be achieved in two steps: first, by sorting the detected persons in the tracking step, for example, by triangulating all the persons whose reprojection error is below a certain threshold instead of taking only the one with minimum error, in a similar way as carried out by Slembrouck et al. [48]; then, by tracking the triangulated persons in time, e.g., by limiting the displacement speed of each person’s neck keypoint from one frame to the next one. 

Both the 2D pose estimator model and the OpenSim models are of crucial importance. The choice of the OpenPose Body_25b model gives better results than the default Body_25 one; however, they both only detect 25 keypoints. This makes it hard for OpenSim to solve internal/external rotations around limbs, as well as angles of the spine and of the lombo-sacral joint. Additionally, there is no marker for the hand, which makes it impossible to render any pronation/supination. Using the experimental Body_135 OpenPose model would solve this issue, although it would also increase the computational cost. Another way of carrying this out would be to train a custom model with DeepLabCut. Although it requires time and energy to label a large dataset of human beings, it could give the opportunity of helping the OpenSim global optimization by using several markers per joint, in the same way as markersets are designed in marker-based methods.

Furthermore, the OpenSim model needs to be carefully crafted. Inverse kinematics is an under-constrained problem that can be guided with carefully chosen joint constraints. The placement of markers on the model is also of paramount importance, especially with so little of them. OpenPose keypoints do not necessarily coincide with joint centers, and they may be located in a different anatomical position when limbs are fully extended in comparison to when they are fully flexed. However, once the markers are positioned in accordance with those of the 2D pose estimations, the scaling step is very fast and straightforward since the marker placement will not change from one session, subject, or operator to another. Moreover, the Rajagopal model [69] used as a basis does not appear to be well suited for road cycling since the spine is designed as a single rigid bone that cannot bend. This results in sharp shifts in hip abduction/adduction and internal/external rotation angles, which are not biomechanically coherent (see Appendix B Figure A2). However, the same issue would prevail even with marker-based methods and would only be solved by changing the spine definition in the OpenSim model. The full-body model with a fully articulated spine and ribcage developed by Bruno et al. [78] has far too many degrees of freedom for our small amount of detected keypoints; however, the lifting full-body (LFB) model validated by Beaucage-Gauvreau et al. [76] solved the spine challenge by constraining vertebras movements to each other, without adding any degrees of freedom. It would be interesting to combine this model to the Rajagopal one in order to benefit from best accuracies both in the knee and in the spine kinematics.

Desmarais et al. proposed a taxonomy of 3D pose estimation algorithms based on accuracy, speed, and robustness [59]. Although more modalities could be tested, our workflow was robust in the range of our tested conditions. Speed was unaddressed, but although real-time analysis seems out of reach, a timely analysis of athletes’ movements directly on the sports field appears to be achievable. Indeed, OpenPose is faster than most of its competitors (see Chen et al. [23]), and the rest of the process (i.e., estimating pose, tracking the person of interest, triangulating, filtering, and solving inverse kinematics) is not computationally costly. Yet, ultimately, the accuracy of the workflow must be concurrently validated with a reference marker-based method. This will be the topic of a future article.

## Figures and Tables

**Table 1:** Descriptive statistics on the triangulation step performed by Pose2Sim from OpenPose body_25b model. Mean absolute reprojection error and mean number of excluded cameras were calculated over time. Mean (mean), standard deviation (std), and maximum (max) in each of these variables are displayed. Walking, running, and cycling tasks were investigated in each four conditions: reference (Ref), poor image quality (Im), four cameras instead of eight (4c), and calibration errors (Cal).

Tasks	Conditions	Mean Number of Excluded Cameras			Mean Absolute Reprojection Error		
		Mean	std	Max	Mean	std	Max
Walking	Ref	0.47	0.57	2.0 (Nose)	3.3 px (1.6 cm)	1.1 px (0.54 cm)	5.3 px (2.6 cm, LHip)
	Im	0.91	0.80	2.4 (LWrist)	3.7 px (1.8 cm)	1.0 px (0.52 cm)	5.2 px (2.6 cm, LSmallToe)
	4c	0.27	0.34	1.0 (Nose)	2.9 px (1.4 cm)	0.93 px (0.47 cm)	4.5 px (2.2 cm, LSmallToe)
	Cal	0.47	0.57	2.0 (Nose)	5.1 px (2.5 cm)	0.91 px (0.45 cm)	6.9 px (3.4 cm, LHip)
Running	Ref	0.48	0.64	2.2 (LWrist)	3.5 px (1.7 cm)	1.2 px (0.57 cm)	5.6 px (2.8 cm, LWrist)
	Im	0.94	1.2	4.5 (LWrist)	4.0 px (2.0 cm)	1.4 px (0.69 cm)	7.2 px (3.6 cm, RWrist)
	4c	0.22	0.31	1.0 (LWrist)	3.3 px (1.6 cm)	0.97 px (0.48 cm)	4.7 px (2.3 cm, LWrist)
	Cal	0.47	0.65	2.2 (LWrist)	5.4 px (2.7 cm)	1.0 px (0.52 cm)	7.2 px (3.6 cm, LWrist)
Cycling	Ref	1.62	1.4	4.2 (RBigToe)	6.1 px (3.0 cm)	1.2 px (0.58 cm)	8.5 px (4.2 cm, Head)
	Im	2.41	1.9	5.7 (RBigToe)	6.3 px (3.1 cm)	1.3 px (0.60 cm)	8.5 px (4.2 cm, Head)
	4c	0.76	0.67	2.1 (RBigToe)	5.3 px (2.6 cm)	1.6 px (0.82 cm)	8.4 px (4.2 cm, LElbow)
	Cal	1.68	1.4	4.24 (RBigToe)	6.9 px (3.4 cm)	1.0 px (0.51 cm)	8.9 px (4.4 cm, Head)

**Table 2:** Descriptive statistics on the inverse kinematics step performed by OpenSim with a full body model adapted from Rajagopal’s [69]. Root mean square (RMS) errors between experimental and model markers were calculated over all markers. Mean, standard deviation (std), and maximum (max) are displayed. Dead center refers to the phase where the crank is near the vertical position.

Tasks	Conditions	RMS Marker Error		
		Mean	std	Max
Walking	Ref	2.8 cm	0.13 cm	3.2 cm (Mid stance)
	Im	2.8 cm	0.11 cm	3.1 cm (Mid stance)
	4c	2.8 cm	0.12 cm	3.2 cm (Mid stance)
	Cal	2.9 cm	0.13 cm	3.2 cm (Mid stance)
Running	Ref	2.2 cm	0.22 cm	2.6 cm (Mid stance)
	Im	2.4 cm	0.21 cm	2.8 cm (Mid stance)
	4c	2.5 cm	0.30 cm	2.4 cm (Mid stance)
	Cal	2.2 cm	0.21 cm	2.6 cm (Mid stance)
Cycling	Ref	3.4 cm	0.11 cm	3.6 cm (Dead center)
	Im	3.8 cm	0.18 cm	4.2 cm (Dead center)
	4c	3.9 cm	0.60 cm	5.9 cm (Dead center)
	Cal	3.4 cm	0.11 cm	3.6 cm (Dead center)

**Table 3:** Summary of angles statistics, averaged for all joints. Each condition is represented: reference condition (Ref), degraded image quality (Im), four cameras instead of eight (4c), degraded calibration (Cal). Comparisons between each Im, 4c, Cal conditions, and Ref are accounted for with standard deviation (std), the standard deviation ratio (std/std_ref_), the Pearson’s correlation coefficient (r), and the mean absolute error (MAE).

Task	Conditions	std (°)	std/std_ref_	r	MAE (°)
Walking	Ref	2.56	-	-	-
	Im	3.03	1.19	0.97	1.55
	4c	3.24	1.27	0.97	1.50
	Cal	2.60	1.02	1.00	0.35
Running	Ref	2.59	-	-	-
	Im	2.76	1.07	0.99	0.92
	4c	2.79	1.10	0.97	1.60
	Cal	2.54	0.98	1.00	0.47
Cycling	Ref	1.78	-	-	-
	Im	1.89	1.08	0.88	1.72
	4c	3.04	1.93	0.81	1.54
	Cal	1.80	1.02	0.99	0.50
Cycling (lower-body only)	Ref	2.09	-	-	-
	Im	2.41	1.22	0.94	1.69
	4c	3.82	2.31	0.90	1.84
	Cal	2.13	1.03	0.99	0.51

**Table 4:** Stride-to-stride standard deviations of lower-body angles, with a comparison between the markerless approach of the current study and a marker-based one (averaged over 18 young subjects) [72]. * Ankle subtalar angle is assimilated to an abduction/adduction angle.

Joint	Method	Flexion/Extension	Abduction/Adduction *	Internal/External Rotation
Ankle	Kang et al. [72]	2	2.5	-
	Ours	2.07	4.84	-
Knee	Kang et al. [72]	0.7	-	-
	Ours	4.85	-	-
Hip	Kang et al. [72]	1.2	1.8	1.1
	Ours	2.61	1.5	3.72

**Table 5:** Summary of angles statistics in the three rotation planes, averaged over all joints (n = 5, 3, 2 for Flexion/Extension, Abduction/Adduction, and Internal/External Rotation, respectively.). All conditions are represented: degraded image quality (Im), four cameras instead of eight (4c), and degraded calibration (Cal). These conditions were compared to the reference one (Ref) by calculating the ratio of standard deviation (std/std_ref_), the Pearson’s correlation coefficient (r), and the mean absolute error (MAE). * Ankle subtalar angle is assimilated to an abduction/adduction angle.

		Flexion/Extension			Abduction/Adduction *			Internal/External Rotation		
		std/std_ref_	r	MAE (°)	std/std_ref_	r	MAE (°)	std/std_ref_	r	MAE (°)
Walking	Im	1.11	1.00	1.48	1.28	0.98	1.05	1.23	0.89	2.47
	4c	1.15	1.00	0.90	1.29	0.93	1.28	1.54	0.94	3.35
	Cal	1.01	1.00	0.19	1.04	0.99	0.50	1.04	0.99	0.54
Running	Im	1.03	1.00	0.98	1.08	0.98	0.48	1.13	0.98	1.41
	4c	1.03	1.00	0.98	1.18	0.93	1.00	1.14	0.97	4.06
	Cal	1.00	1.00	0.30	0.99	0.99	0.53	0.92	1.00	0.80
Cycling	Im	0.99	0.97	1.89	1.27	0.66	1.45	0.99	0.97	1.71
	4c	1.31	0.96	1.43	3.10	0.46	1.76	1.71	0.97	1.47
	Cal	1.00	1.00	0.39	1.06	0.96	0.36	1.02	1.00	1.00

## Data Availability

Data and code will be provided upon publication of the article.

## Supplementary Materials

The following are available online at https://www.mdpi.com/article/10.3390/s21196530/s1, Video S1.
